# Reducing the indication of ventilatory support in the severely burnt patient and improving outcomes: Results of a new protocol approach within a regional burns centre

**DOI:** 10.1186/cc11073

**Published:** 2012-03-20

**Authors:** J Gille, H Taha, R Blankenburg, T Raff, A Sablotzki

**Affiliations:** 1 St Georg Hospital, Leipzig, Germany; 2 Royal Devon & Exeter Hospital Foundation Trust, Exeter, UK

## Introduction

Initial management of the severely burnt patient often includes sedation and mechanical ventilatory support as routine. Conversely it is documented in the literature that nonjudiciously applied mechanical ventilatory support can itself lead to poorer patient outcomes [1]. Exploring means to reduce this iatrogenic risk, a standardised in-house five-point protocol offering clinical guidance on the use and duration of ventilation was introduced, analysed and the impact on outcome assessed.

## Methods

A clinical observation study, approved by the local ethical committee, was designed and executed. Criteria for early spontaneous breathing were defined. These were formulated into a protocol for the management of severely burnt patients and trialled over 2 years in clinical practice on all admitted patients (group A). The ventilation period, complications and final outcomes were recorded and compared with a retrospective control group of patients (group B) collated prior to implementation of the protocol. Initial study analysis revealed high inclusion rates of superficial burns in the intervention group. To achieve comparability these were excluded and further analysis was conducted only for patients with an abbreviated burn severity index (ABSI) ≥7.

## Results

In total 118 patients were included. The demographics and injury characteristics of both groups were similar. Patients of group A (*n *= 61) had fewer ventilator days in the time course of treatment (3.9 ± 11.7 vs. 17.1 ± 19.6 days, *P *< 0.01). Affiliation to group A correlated with a shorter time of ventilation after admission (*P *< 0.01); 61.1% of these patients were extubated within 6 hours after admission (vs. 14.3% in group B). Group A showed lower mortality rates (1 (1.4%) vs. 8 (14%), *P *= 0.01), shorter total hospital stay (34.2 ± 23.9 vs. 50 ± 38.4, *P *= 0.014) and lower incidence of sepsis (24 (39.3%) vs. 39 (68.4%), *P *< 0.01). No patients fulfilling the inclusion criteria required re-intubation or emergency intubation.

## Conclusion

Extended periods of mechanical ventilatory support are known to be associated with poorer outcomes in the severely burnt patient. Guidance on minimising ventilator dependency through introduction of a protocol has led to improved outcomes of such patients within a regional burns centre. This study suggests that many burns patients are overtreated through routine ventilation.
